# Exploring the potential of a school-based online health and wellbeing screening tool: professional stakeholders’ perspectives and experiences

**DOI:** 10.1186/s12889-022-12748-2

**Published:** 2022-02-16

**Authors:** Nicholas Woodrow, Hannah Fairbrother, Katie Breheny, Katrina d’Apice, Patricia N Albers, Clare Mills, Matthew Curtis, Lisa Hopkins, Sarah Tebbett, Rona Campbell, Frank De Vocht

**Affiliations:** 1 School of Health and Related Research (ScHARR), University of Sheffield, Regent Court, S1 4DA Sheffield, UK; 2 Health Sciences School, University of Sheffield, 3a Clarkehouse Road, S10 2HQ Sheffield, UK; 3 Population Health Sciences, Bristol Medical School, Barley House, University of Bristol, Oakfield Grove, Clifton BS8 2BN Bristol, UK; 4 Public Health, Floor 4, Halford Wing, City Hall, 115 Charles Street, LE1 1FZ Leicester City Council, UK; 5 Leicestershire Partnership NHS Trust, Bridge Park Plaza, Bridge Park Road, Thurmaston, Leicestershire LE4 8PQ Leicester, UK

**Keywords:** School, Health screening, Children and young people

## Abstract

**Background:**

Supporting children and young people’s (CYP) mental and physical health is a global policy priority but detecting need and facilitating access to health services and support is challenging. This paper explores professional stakeholders’ perspectives of the acceptability, utility and effectiveness of a school-based online health and wellbeing screening tool, the Digital Health Contact (DHC). The DHC, delivered by Public Health School Nurses (PHSN), aims to identify, and put in place strategies to support, unmet health needs among CYP.

**Methods:**

We employed a qualitative study design, using semi-structured interviews. Fourteen key stakeholders involved in the design and implementation of the DHC (commissioners, providers, PHSN and healthcare staff, school leaders) were purposively sampled. Data were analysed thematically.

**Results:**

Our analysis generated two key themes: the perceived benefits of the DHC; and challenges in delivering the DHC. Stakeholders perceived the universal application of the DHC with linked follow-up intervention as an effective means of identifying and supporting CYP with unmet needs, and an efficient way to target limited service resources. There were barriers around enabling school engagement in the DHC, typically in terms of logistics, school infrastructure, and perspectives of fit with schools. These barriers were seen as being negated through developing effective working relationships between schools and PHSN. Effective relationships could highlight the potential benefits of participation. Overall, the DHC was seen as a valuable and effective use of resources, with a low burden on school staff.

**Conclusions:**

The DHC, as a universal school-based health and wellbeing screening tool with linked follow-up intervention, has great potential in identifying and supporting unmet health needs among CYP. The perspectives and experiences of those involved in delivering the DHC highlight important considerations which may enable effective implementation and delivery of school screening programmes across other areas.

**Supplementary Information:**

The online version contains supplementary material available at 10.1186/s12889-022-12748-2.

## Introduction

Supporting children and young people’s (CYP) mental and physical health is a global policy priority [1, 2]. In the UK, the National Health Service’s (NHS) Long Term Plan [3] sets out action to improve the health and wellbeing of CYP aged 0-25. Whilst there is a welcome recent reduction in the prevalence of some health risk behaviours in CYP in the UK (notably in tobacco, alcohol and drug use [4]), there is evidence that the prevalence of mental health disorders is increasing [5]. Indeed, estimates suggest that at least one in six CYP in the UK aged 5-16 have a mental health condition [5]. However, these estimates may represent just the ‘tip of the iceberg’, with some studies showing much higher levels of mental health problems among CYP, associated with gender, deprivation, ethnicity and age [6]. Worryingly, the Covid-19 pandemic has had a considerable adverse impact upon CYP health and wellbeing [5], alongside concerns around continued and undetected abuse and exploitation of CYP [7, 8]. It is well established in the literature that many CYP who experience health and wellbeing issues do not access support [9, 10]. Studies highlight a reluctance to access health services due to concerns around perceived stigma of service engagement or support seeking [11], perceptions that services may not be appropriate for their needs, accessible or be able to help [11, 12], and a reliance on informal avenues of support (e.g., friends) [11, 13] among CYP. This is important since early intervention and support are consistently associated with better outcomes for people who experience health and wellbeing issues [14, 15]. One potential avenue for early identification of and support for CYP with physical and mental health needs is through schools [16, 17]. As many mental health problems which persist into adulthood develop during adolescence [18], the near universal and consistent contact schools have with CYP [19, 20] highlights the benefit, importance and opportunity schools have in early detection and intervention around physical and mental health needs for CYP.

### Schools as a potential avenue for the detection of unmet need among CYP

While staff in schools are in an opportune position to identify needs among CYP, this has been shown to be problematic [15, 21, 22], especially for internalising disorders (such as depression, anxiety, suicidal ideation) [14, 23, 24] and safeguarding concerns [21, 22]. School staff have been found to miss and under-identify needs in young people [14], and, consistent with this, have reported struggling with and feeling unprepared in identifying mental health needs in pupils [15]. Detecting physical and mental health need is often based upon behavioural or educational risk markers, and often occurs retrospectively following a ‘crisis’. This ‘wait to fail’ model in identification and referral results in both under-referral and late-referral for support [20]. Under-identification is a salient contributor to the gap between CYP’s needs and their support and treatment [9, 19, 25]. The challenges for school staff in detecting physical and mental health needs highlight the importance of alternative routes to identification. One such alternative is school-based screening. There is a nascent literature around online, self-report, school-based screening surveys which suggests such approaches may increase accessibility of support services for CYP [12, 14, 26]. School-based screening could be offered alongside existing routes - CYP proactively arranging a meeting with school staff members, and the ad-hoc identification of health issues by school staff based on overt risk identifiers [19, 20, 27]. Indeed, online and electronic screening tools have been associated with CYP disclosing sensitive information without fear of being judged, and leading to more disclosures [28–30]. In this way, when undertaken appropriately, screening programmes in schools may be an effective and accepted tool for identifying risk, and a tool which may not cause undue distress for CYP [15, 29]. Screening and associated follow-up support may have beneficial outcomes in enabling those with treatable health conditions to be identified at an early stage, thus lessening the adverse impact of health conditions. Using surveys as a screening tool to identify students with unmet needs, therefore, has the potential to be an effective use of resources. However, there is currently limited evidence around the effectiveness, feasibility, and acceptability of school-based screening [14, 15, 20, 26].

Our study addresses this gap as we evaluate a novel, multi-stage health and wellbeing screening and intervention programme, the Digital Health Contact (DHC). The DHC was initially piloted in 2017 and has been running since then in the East Midlands of England. Below we describe the DHC, drawing upon the Template for Intervention Description and Replication (TIDieR) checklist and guide [31].

### The Digital Health Contact (DHC)

**Fig. 1:**
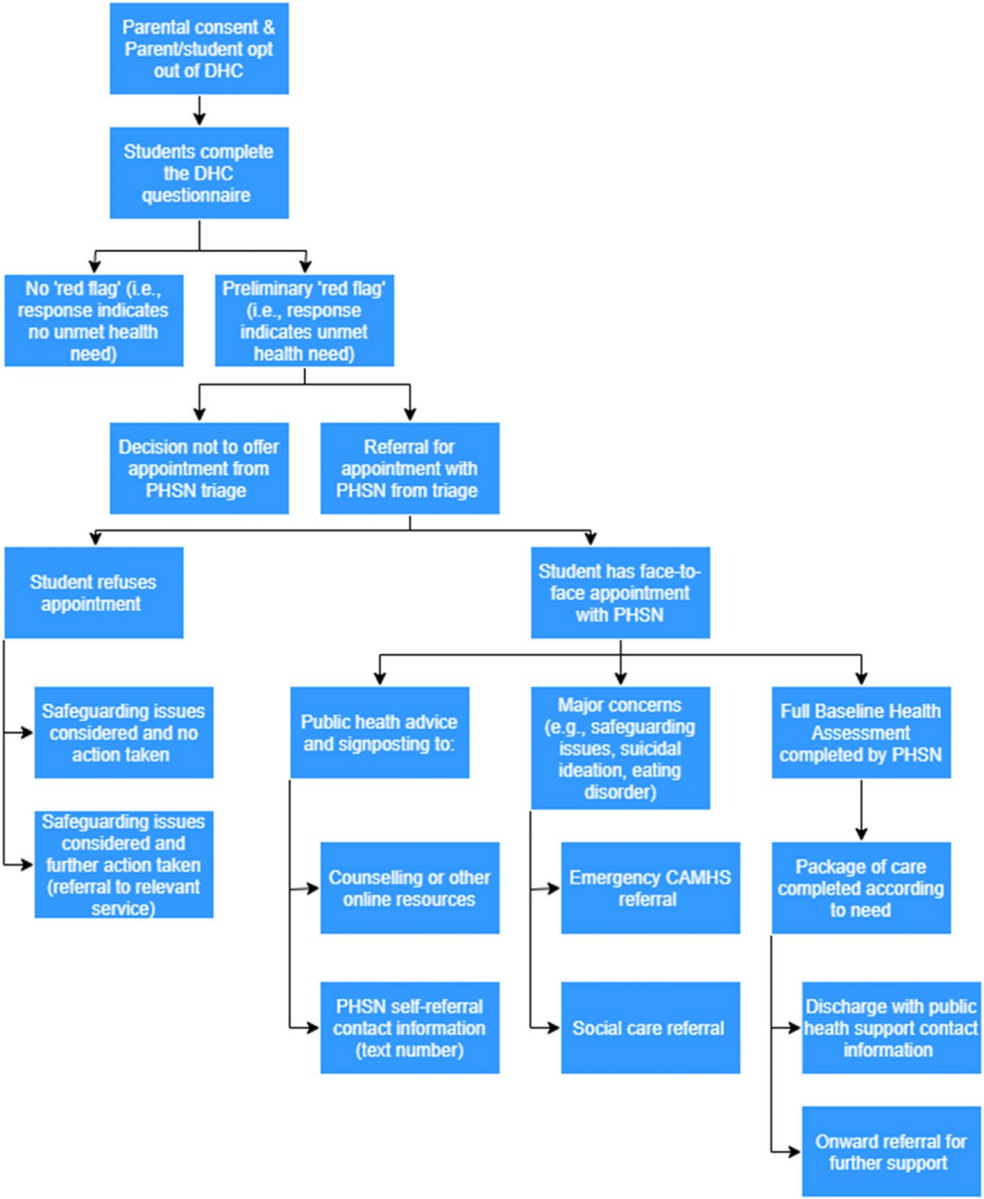
DHC flowchart

The DHC is commissioned by Leicester City Council (LCC) as a non-mandated part of the 0-19 Healthy Child Programme (HCP), with Leicestershire Partnership NHS Trust (LPT) as the provider. The DHC is an online questionnaire completed by an entire secondary school year group (currently running in year 7 (aged 11-12), and year 9 (aged 13-14)). It consists of 30 questions around physical and mental health and wellbeing, covering a range of topics including self-harm, mood, body image, diet, substance use, and sexual health (see Additional file 2 for a full list of questions and topics covered). All questions are closed, with the option for qualitative responses for further information. The DHC acts as a universal screening tool, with indicated face-to-face intervention and follow-up from Public Health School Nurses (PHSN) for those among whom unmet needs are identified. The DHC aims to provide an immediate method for identifying CYP with unmet needs, and allows evidenced based support to be offered and put in place by PHSN (see ‘Fig. 1 - DHC flowchart’, for an overview of the DHC screening process).

Rather than adopting questions from validated tools, the scope and questions in the DHC were devised by the provider organisation (including public health team leaders and clinical staff) with overview and comment from commissioners. A key aim was to cover a breadth of health and wellbeing topics in a concise tool to reduce time burden on schools, and therefore encourage their participation. Questions to identify unmet needs were devised from key ‘risk’ themes based around local policy and prevalent clinical issues for CYP’s physical and mental health. Recognising the limitations of not using validated measures [32], no existing tool was deemed suitable in covering the scope of physical and mental health and wellbeing topics required by the commissioning organisation, whilst also being concise enough to fit around time and resource considerations of the participating CYP, schools and providers. During the DHC development stage, focus groups with CYP across a range of ages (11-16 years old) were carried out to explore their perspectives of the questions, the wording of the questions and perceived understanding. This led to some reshaping of questions and terminology to ensure comprehension.

PHSN are responsible for recruiting schools to participate in the DHC. Participation is optional and negotiated on an academic year-by-year basis. Pupils are provided with an overview of the DHC, typically in a school assembly given by the PHSN, and parents/guardians are sent an information letter detailing the DHC with the option to opt their child out. All CYP have the right to decline to take part in the DHC and are required to opt-in to participate. Following acquiring consent from CYP, teachers facilitate the questionnaire using school computers during class time, with a whole year group taking part over one to two days. Responses are processed automatically, and all CYP are provided with a digital personalised care plan upon completion of the questionnaire; the CYP can save this for later use or to discuss with parents/guardians or other support options. The plan contains generic public health advice, but can also include signposting, advice and support based upon the answers provided. The wording of the advice and support information in the care plan is the same for all age groups and was refined in focus groups with CYP to maximise comprehension.

Certain responses or words/phrases given by participants produce a ‘red flag’ alert on their health records system. If a child ‘red flags’ by showing unmet health need, a referral alert is sent to the PHSN team. These referrals are triaged by a PHSN who will contact any CYP deemed at risk/to have unmet need to offer a face-to-face initial health assessment in school. Appointments for initial assessments are made within two weeks of triage, with those posing the highest risk being prioritised (e.g., self-harming, low mood, and safeguarding concerns).

CYP are removed at the triage stage if PHSN do not deem them to have unmet health need or if CYP records show that they are already involved with a specialist service. CYP can be sent relevant public health information via letter or email. Any CYP offered an initial assessment has the right to decline, with this then being followed up by school pastoral staff. Initial assessments typically last 20 min and the outcome can be signposting and delivery of advice/digital resources, an urgent referral to a specialist service (CAMHS, Social Care) or a full Baseline Health Assessment (a comprehensive holistic assessment developed by LPT and delivered by the PHNS, which typically lasts 60 min, covering all aspects of physical, social and emotional health). From the Baseline Health Assessment, a relevant, evidence-based package of care may be implemented – this typically lasts four sessions and is delivered by a PHSN or health practitioner. Following this, PHSN assess whether the CYP requires further support, referral to a specialist service or if they will be discharged.

### Research aims

Informed by a realist evaluation framework [33], this paper explores key stakeholders’ perspectives on the effectiveness and acceptability of the DHC in identifying and putting strategies in place to meet unmet health needs of CYP. Key stakeholders comprised those involved in the delivery of the DHC programme (providers and commissioners, PHSN and practitioners delivering the programme, school leaders). Subsequent papers will report on the perspectives of CYP who have participated in the DHC, and whether the DHC has increased referrals to the PHSN and in turn improved the health and wellbeing of the young people referred.

## Methods

### Sample and recruitment

Fourteen key stakeholders involved in the design and implementation of the DHC were purposively sampled. The commissioner and main provider facilitated recruitment of participants via an initial email invite. Sampling aimed to include up to four schools participating in the DHC during data collection (seeking to work with schools reflecting a maximum diversity in terms of urban/rural location, affluence/deprivation, ethnic diversity and faith schools and single gender schools). Intended participants included one commissioner and one provider, and for each school, the school lead for the DHC, two PHSN delivering the DHC, one Healthy Child Programme Practitioner or Support Worker delivering the DHC support. However, data collection began in Autumn of 2020, in the midst of the Covid-19 pandemic, which negatively impacted upon recruitment, as schools were shut and the DHC programme paused. The final sample consisted of two commissioners, one provider, three school leaders, six PHSN and two Healthy Child Programme Practitioners. Despite challenges in recruitment, we were able to recruit school leaders and professional stakeholders working in urban and rural (city and county) locations of higher and lower deprivation.

### Ethics

Ethical approval for the study was granted by the School of Health and Related Research (ScHARR) ethics committee at the University of Sheffield. All participants provided written informed consent for their involvement in the research.

### Data collection

Due to Covid-19 lockdown restrictions and social distancing requirements, in-depth semi-structured interviews were undertaken via online video call platforms, or through telephone interviews. Interviews were facilitated by NW. All interviews lasted between 30 and 60 min. All participants electronically signed and returned a consent form before participation. The interviews followed semi-structured topic guides (Additional File 1) which explored perspectives and experiences of the programme, focusing on understandings of the DHC, schools’ perspectives for participation, priority outcomes, implementation and delivery issues, capacity to deliver the programme, process reflections, strengths and weaknesses, and future plans. Effort was actively made to ensure all participants were asked all questions in the topic guides. If the questions/topics were not organically covered during the semi-structured interview, they were explicitly asked. The interviews tended to flow smoothly, enabling the questions to be asked in the order of the schedule, but where other topics emerged naturally during the conversation these were explored. All interviews were audio-recorded using an encrypted recorder, transcribed verbatim by a third-party transcription company, anonymised at the point of transcription, and checked for accuracy by NW.

### Data analysis

Interview data were analysed by NW and HF drawing on Braun and Clarke’s thematic analysis approach [34]. An initial coding framework was developed based on the interview topic guides and questions. Both NW and HF read and coded a selection of transcripts from all participant groups (providers, commissions, school leaders, and PHSN). The coding framework was then revised following discussions between NW and HF. Additional initial codes were added, and then the codes were examined, merged, and grouped into potential themes and sub-themes. The revised framework (additional file 3) was applied to all the transcripts by NW, and HF separately coded a selection of transcripts to check for accuracy and consistency. Codes were assigned to each response (multiple codes were allocated to each response if required). NW and HF reviewed and refined the themes. Transcripts were coded using the qualitative data management software system NVivo-12.

## Results

Two overarching themes emerged from the data: ‘The Perceived Benefits of the DHC’ and ‘Challenges in Delivering the DHC’. These themes, and linked sub-themes, are discussed below (see Table 1 for overview).

**Table 1:** Themes and sub-themes overview

Theme	Sub-theme
The Perceived Benefits of the DHC	- Detecting (unmet) health need - Promoting awareness and encouraging use of support options - Informing delivery of support systems
Challenges in Delivering the DHC	- Perceived feasibility of the DHC - Time/resource implications - Highlighting the potential benefits of the DHC - Effective relationships and experience of encouraging participation

### The Perceived Benefits of the DHC

#### Detecting (unmet) health need

All participants were positive about the DHC programme and its perceived effectiveness in identifying and providing support for CYP who have not previously been identified as needing support. Many of the workers involved in delivering the DHC programme could offer numerous examples of how the DHC screening had helped identify CYP with significant unknown and undiagnosed health issues (e.g., eating disorders, self-harm, suicidal ideation):

‘*it really has actually managed to pick up some quite complex sort of needs and cases. And you know, without it, without that contact, then you know, those cases might not have actually been picked up*’ (ID8 Commissioner).

This perceived success drove a desire from practitioners to recruit more participating schools. Whilst the benefits of engagement for all the school leaders encouraged continued participation: ‘*now that we’ve had it, I wouldn’t want to lose it*’ (ID13 School Leader).

A major perceived benefit of the DHC was its universal application and linking of responses to individual students. This was seen as giving the opportunity to hear the voices of those not typically identified and apparent to services, and providing needed follow-up support to these CYP. The approach of providing questions and additional information across various topics was also seen as helping those who may not realise they need support, or whose issues were ‘normalised’. There was a belief among the PHSNs that the DHC referrals were qualitatively different from those of existing referral sources (e.g., school or self-referral), and identified CYP not previously known to services:

‘*I’ve picked children up that have had no support in the past, not even told their parents, schools, anybody. So we are picking up young people that otherwise would have sort of maybe continued to self-harm and, you know, just escalated further*’ (ID10 PHSN).

The managing of the programme by PHSN was also seen as encouraging more honest responses as their role was perceived to be separate to that of the school: ‘*I think they’ll always think that we’re judging them as teachers – not that we are but I think they’re more likely to be more honest with an independent person*’ (ID13 School Lead). Due to such perceived benefits, the DHC was seen as a useful complementary tool to aid detection of need:

‘*especially for a student who finds it very difficult to speak to an adult, but wants to speak to an adult, and there’s a cry for help but doesn’t know where to go. You need many different platforms to do that in, and this is one*’ (ID14 School Lead).

#### Promoting awareness and encouraging use of support options

The DHC was seen as effective in helping to raise awareness among CYP that they can access support through PHSN, as well as highlighting other support options. It was also described as helping to build positive relationships between pupils, PHSN and schools. An important outcome noted by several PHSN was a perception of increased awareness of PHSN identity and role, among both students and school staff. The increased face-to-face contact and exposure to students the DHC provides was seen as helping to promote the PHSN service and highlighting the wider PHSN offer:

‘*I’ve noticed doing the questionnaires, the teenagers were much more aware of who I was within the school. When you’re walking around they know who you are, you’re the school nurse and things like that. So in a way it’s very good to promote our service as well because we’re doing the assemblies and things but because you see quite a large group of young people*’ (ID12 PHSN).

This increased awareness was also noted to have encouraged students to contact PHSN for issues experienced outside of those discussed in the DHC:

‘*I’ve had young people come back for different things, not what put them on the red flag, it can be a few months later, they’ll come back because they want to talk to you about something else that’s cropped up, another worry that they’ve got, so they know you’re there and they trust you… that’s a good thing about these questionnaires, it’s not just at that time, it’s like as they get to know you they’ll come back and see you about other things*’ (ID11 PHSN).

Such examples were used to highlight that the right messages around signposting support are being delivered in the right ways, with this increasing accessibility to PHSN, and confidence from CYP to engage with them.

#### Informing delivery of support systems

Once set up and running in schools, the DHC was perceived by all study participants as an extremely valuable tool which provides support for detecting needs at both an individual and population (year, school and regional data/trends) level. The data generated was described as significant in helping schools and PHSN prioritise and respond to the specific issues that year groups and whole schools are experiencing:

‘*you can actually develop an action plan to actually address those needs being identified*’ (ID8 Commissioner).

‘*one of the things we found is about students being anxious, but they didn’t know how to get information or trusted websites…since then we’ve also got a mental health first aider in place as well. So these are some of the spin offs that have come through this*’ (ID14 School Lead).

Data from the DHC enables PHSN to design and deliver bespoke health fairs for schools: ‘*we can tailor the health fairs to make sure that we’re targeting what the young people need*’ (ID6 PHSN). This was seen by the school leads as a more effective use of resources as it enabled support and information to be delivered on issues which the CYP themselves had revealed as prevalent. One school leader noted how the different needs detected among different year groups allowed specific packages of care to be implemented, as well as using anonymised school level data to support work:

‘*what I think is always quite interesting…is how many of them actually have had a sexual encounter. Because they all think everybody is, but when you can say “Actually this is what your statistical data shows” it gives them the confidence that they don’t think everybody else is. So I think that sort of data is really important to feed in, because it’s their real dat*a.’ (ID7 School Lead).

### Challenges in Delivering the DHC

PHSN are responsible for recruiting schools to participate in the DHC at the start of each academic year. The PHSN see part of their role as ‘selling’ the DHC to schools, but as one PHSN noted, ‘*obviously we’re not sales people by trade!*’ (ID9 PHSN). The securing of school participation, or the ‘selling’ of the programme to schools, was noted to have some challenges. Hesitancy or reluctance around participation from some schools centred around feasibility and acceptability issues, including, logistical issues (classroom space, IT infrastructure, concerns around school staff time commitments) or perceptions regarding ‘fit’ of the DHC for particular schools (school ethos, perception of value, appropriateness of questions). Those involved in presenting the DHC to schools noted how they had to highlight the benefits of involvement and demonstrate that potential issues had been planned and accounted for. It was suggested by the PHSN that having a document of ‘key points’ to deliver to schools (comprising examples of work, how issues had been overcome, what data schools are provided with and recommendations from other schools who have been involved), would help facilitate a consistent message to all prospective participating schools. It was noted that, for schools, ‘*the more information, the more reassurance they get, then the more likely they are to engage*’ (ID10 PHSN).

#### Perceived feasibility of the DHC

In relation to perceived challenges around feasibility among school leaders, PHSN described how they developed counter arguments to many common hesitancies. The PHSN noted using case studies, not only around their experience of delivering the DHC in large schools with limited IT facilities, but also around detecting and supporting CYP with unmet needs to highlight its value:

‘*So we’ve used case studies quite well to demonstrate to the school the benefits of using it, because what we found…is that often the kids with the best outcomes, they weren’t on anybody’s radar in the first place. So our best case studies are because nobody was concerned about these kids*.’ (ID2 Provider).

The PHSN also set up meetings and directed hesitant schools to schools in which the DHC has been successfully implemented, to facilitate learning and encourage participation: ‘*They [PHSN] pointed out to schools that have managed it, and then I went over to those schools to see what did they do, just to model that back into our schools*’ (ID14 School Lead). There were, however, more challenging issues for school engagement, which were seen as difficult to counter. There were perceptions among PHSN that some schools are less proactive in supporting CYPs health and wellbeing, and thus less likely to participate in programmes like the DHC. Cultural differences were also noted, with a suggestion that some schools (typically faith schools) were not comfortable in questions around specific topics (e.g., sexual health):

‘*So sometimes we have difficulties with our faith schools, so some of the faith schools in the city haven’t wanted to take part because they haven’t been in agreement with some of the questions in the form*’ (ID2 Provider).

#### Time/resource implications

In terms of time and other resource implications for schools, it was noted by PHSN and school leaders, that as the PHSN manage the DHC data and deliver the work following the set-up and implementation of the questionnaire in classes, the burden on school staff is low. PSHN thought that the low burden on schools was a major incentive for their engagement. This was echoed by the school leads who noted that whilst the initial setup of the programme can be more resource intensive, once up and running, it has minimal and manageable impacts on school staff time: ‘*Once I’ve set it up and once you know what you’re doing, it’s not too bad…it’s just really admin time but for the teachers it’s not a big issue at all*’ (ID13 School Lead).

Having an invested and influential key contact point in each school who would organise the logistics of implementation and follow-ups in the school (e.g., booking rooms, arranging IT support for when the classes would undertake the questionnaire) was noted as being crucial for the DHC (and for promoting PHSN services more generally). Both PHSN and school leads asserted that having ‘the right contact’ facilitated effective engagement:

‘*I’ve got a key person in school and she’s really good, but she is the person that organises it all from start to finish…I’ve been quite lucky and fortunate that I’ve got one person that I deal with for the whole process within the school*’ (ID11 PHSN). ‘*I would say that the person in charge of it in a school needs to be a member of SLT [Senior Leadership Team]…Now of course they’re the busiest people, but they are of course are the ones who can make things happen more effectively as well. I would say that it should be organised by a senior leader*’ (ID14 School Lead).

In relation to time/resource implications for those delivering the DHC, practitioners reported initial interest and excitement when presented with the programme, but apprehension around the potential increase in their already heavy workload [35]. This apprehension was noted to lower once they began delivering the programme and saw that the additional work was not as intensive as initially believed and that this work aligned with current roles. In addition, due to the structured approach of the DHC programme (school participation was planned in advanced and arranged across an academic year), any additional work was deemed manageable, and an effective use of their time. Many PHSN saw the pupil-focused contact as a meaningful part of their role, and the work they *should* be doing being a PHSN:

‘*you get to go and see lots of kids who we wouldn’t necessarily have seen before and do – it’s what I would call proper Public Health work – the stuff that we’re supposed to do – you know, we get to go and see the kids and give them advice and signposting and, albeit it brief, ten minutes isn’t very long, but it feels like it’s what we should be doing*.’ (ID2 Provider).

Practitioners acknowledged that some CYP were being seen that did not need to be seen due to either misinterpretation of questions, or them no longer expressing/experiencing the same issues as when they had completed the questionnaire. However, only a small number of false positive cases were noted to be seen at an initial assessment. This resulted in some short triage appointments, but the PHSN noted how this balanced out some of the longer and complex cases, and allowed time for paperwork to be completed. There was a general perception that it was worth seeing CYP to ascertain if they needed support, with the benefits of face-to-face contact outweighing any time commitments: ‘*I’d rather see 200 children through triage if I managed to capture that one person who needed our help*’ (ID4 PHSN). Overall, the DHC was perceived as an efficient way to deliver a universal contact and public health information to a large number of CYP:

‘*we needed a contact for those children in those age groups, we don’t have enough nurses to do that face-to-face, so it was a way of having a universal contact that was offered to all the kids that fit with our staffing models really.*’ (ID2 Provider).

##### Highlighting the potential benefits of the DHC

Emerging programme data and example data reports were used by PHSN to show the DHC’s utility, the benefits to participation for pupils, and what schools gain from participation (e.g., lesson planning and organising of resources around identified needs). Some PHSN noted that schools expressed concerns around the findings of the surveys in respect to Ofsted inspections (i.e., revealing significant levels of need). To counter this, the DHC was presented as a way for schools to demonstrate consideration of pupil wellbeing, and a way for schools to be active in detecting and providing support. It was also highlighted how findings from the DHC could be used to feed into the schools’ Personal, Social, Health and Economic (PSHE) provision, enabling focus on identified school level prevalent issues.

The difficulty of providing robust evidence of value (due to the recent development of the programme) was seen to be a significant challenge in securing school engagement. PHSN described how momentum had begun to build year on year, which encouraged school participation. However, Covid-19 had added further challenges here. For example, schools described as passionate about the DHC were sometimes unable to prioritise the DHC during lockdown restrictions (outside agencies were not permitted in schools due to Covid-19 regulations), and running the programme virtually made the process more complex. However, PHSN noted that some schools were keen to take part in the 2020/21 academic year as the DHC afforded the opportunity to capture CYP’s mental and physical health at a particularly challenging time. There was a universal appreciation that Covid-19 will have an impact upon CYP’s health and wellbeing, and schools will be the best place to detect this and deliver this support. Many of the participants spoke of the DHC being more valuable now than ever:

‘*I think particularly in terms of the impact of Covid as well, particularly on you know, young people’s mental health, I think schools will actually appreciate and realise that they absolutely have to be concerned with the health and wellbeing of children and young people attending their school*’ (ID8 Commissioner).

School leaders described an increased sense of responsibility in managing CYP health and wellbeing, and valued the DHC in helping schools to do so:

‘*I think that happened with the austerity cuts and therefore just less services in the community, not just through the school nurses, but every agency. So there is more pressure on the schools to pick it up and help manage it*’ (ID7 School Lead).*Effective relationships and experience of encouraging participation*.

All PHSN described the importance of effective working relationships between schools and PHSN in facilitating engagement in the DHC. They also highlighted that passion, belief and knowledge among PHSN when presenting the programme to schools as a crucial facilitator in engagement:

‘*it’s going in with a really positive outlook on the questionnaires and actually believing in them as a practitioner… you know, championing the cause of it really, but that’s hard to do without being confident yourself in the process and without having positive experience with it*’ (ID9 PHSN).

The provider noted that ‘championing’ the DHC to schools was challenging for nurses new to the programme. However, experience and understanding of the DHC programme facilitated school engagement: ‘*once your nurses understand it really well, then they can help the school to understand it really well*’ (ID2 Provider).

A Schools initial decision to participate in the DHC was seen to be highly influenced by their relationship with PHSN:

‘*I would be less inclined to use it, if I hadn’t had the school nurse side of it, I probably wouldn’t be using it. And it’s their encouragement of let’s try it again, and we’ll try it this way, and you go and check out this, and their encouragement to keep it going*’ (ID14 School Lead).

In this way, established and effective relationships provided great opportunity and facilitated engagement, permitting the PHSN to more clearly highlight the benefits of participation for each school and their pupils:

‘*I think it’s really important and having that relationship with the school, kind of, it makes it easier to be able to talk about doing the questionnaires or talk about doing other events in school, but also once you’ve got that relationship with them, they kind of trust you a little bit more*’ (ID6 PHSN).

Importantly, a benefit of engagement in the program was also noted to be the fostering of relationships between the schools and PHSN: ‘*The relationship that we’ve had between our school nurse service and us as a school has also improved through this process*’ (ID13 School Lead).

## Discussion

This study has explored the perspectives of professional stakeholders involved in the DHC. Overall, all involved in commissioning, delivering, and hosting the programme highlighted the value and benefits of the DHC programme. All participants described the DHC, and its universal application with linked follow-up support, was perceived as beneficial and effective in identifying and providing support for CYP with unmet physical and mental health needs. The DHC was seen to promote awareness and use of support options. It was perceived as an efficient use of limited service resources and a good way of improving the targeting of service provision - both on an individual level by providing tailored support packages, and on a population level through organisation of school/service resources. There were perceived challenges around the implementation of the DHC programme, typically in terms of feasibility and acceptability issues around logistics, school infrastructure and perspectives of fit with schools. Developing effective relationships facilitated engagement, as did highlighting good practice (previous success and experience of negating implementation issues) and the potential benefits of participation (low time/resource burden for schools, detection of unmet need).

The majority of UK schools report actively attempting to identify CYP with physical and mental health needs [27]. However, most utilise more ad-hoc approaches (e.g., identification through school staff), with there being numerous highlighted challenges and issues for school staff identifying CYP with internalising issues [14, 23, 24]. Highlighting the potential of school-based screening programmes in identifying CYP with health needs [14], the DHC was perceived as improving identification of CYP with unmet health needs. Supporting findings from other school-based surveys which highlight the value of identifying the prevalence of risk factors and needs across a population [27, 36], and echoing previous research looking at screening tools for CYP [37] and mental health screening in schools [15, 38–41], the DHC was discussed as a beneficial tool which aided identification of unmet need, and importantly one that move beyond generically responding to prevalence measures towards effective referral following screening [20]. The DHC was not seen as a panacea or replacement for other approaches for the detecting of unmet health needs in CYP, but as a key tool which compliments the identification of unmet need through providing a universal opportunity for disclosure and advertising services, and an additional avenue to access support through identified follow-up. Indeed, echoing practitioner perspectives of school-based eating disorder screening programme [41], the DHC was perceived as effective in raising awareness of issues and provide information for accessing support.

Further, by producing data at a school, local and regional level, tools like the DHC can enable more effective use of both school and PSHN resources. As well as monitoring the impact of wider factors (e.g. Covid-19), such programmes can help schools to systematically measure wellbeing to plan and deliver interventions (echoing NICE guidance [42]) and help them to meet their requirement to take a proactive approach to identifying and addressing the needs of their pupils [43].

Previous research exploring the feasibility and implementation of school screening programmes have highlighted the resource burden for schools (e.g., staff time, costs) as salient barriers [15, 41, 44–48]. Participation in the DHC, however, has no cost implication for participating schools and this helps to explain why cost was not a concern from the participating school leaders in our study despite being a salient consideration in previous research [44, 45]. Despite time concerns and school staff involvement being discussed as an initial concern in our study, the effective ‘selling’ of the DHC from PHSN helped highlight the limited resource requirements (e.g., staff time) from schools. Indeed, highlighting that PHSN and other health professionals deliver and administer the DHC follow-up sessions was a crucial facilitator in securing participation, as it removed presumed pressure and responsibilities from schools. This supports previous calls for the use of professional agencies to assist schools in identifying health needs and providing follow-up support to reduce engagement barriers [15]. The DHC model may help to obviate concerns around time and resource required to deliver follow-up of identified cases identified in previous studies [41, 46, 48]. Further, and most importantly, effectiveness of interventions for mental health conditions has been shown to be higher when delivered by health professionals than by school staff [12, 49]. Interestingly, though as a barrier for school participation in previous work [39, 47, 50], none of the participants in our study described attaining consent from parents/children as a challenge. This is not to say it was not a concern or an issue, and this finding may be related to our limited sample, but perhaps the process of PHSN being involved in the presentation of programme information to CYP and providing information sheets to schools (see ‘The Digital Health Contact (DHC)’ above) helped negate challenges around consent.

Reflecting findings from other studies, having ‘buy-in’ from key school staff members was a key aspect of feasibility [51], as was effective relationships to help promote uptake and identify and negate challenged/barriers [52]. Our study reinforces the importance of effectively communicating the value of participation to schools [20]. Also, echoing previous work [35, 41, 46, 47], the building and maintaining of productive relationships by PHSN with schools was seen to overcome many barriers around facilitating DHC participation.

As the development of health practices and mental health issues in early childhood are noted to persist into adulthood [53–55] and as support at an early stage may have better outcomes on educational attainment [53, 56] and future treatment (i.e., reduced need [12]), the potential value of universal screening tools such as the DHC is evident. Such tools may permit further identification and support for CYP experiencing and developing need during times of uncertainty and crisis.

### Practical Implications

Effective interventions need to be based upon robust evidence, but they need to consider feasibility and acceptability around logistics, practicalities and implementation issues [15, 51]. Below we discuss key practical implications around the DHC programme facilitation highlighted in this study.

- Acceptability and fidelity of implementation by all parties involved in school screening programmes is crucial (see also [57]). Schools must perceive an intervention as acceptable, feasible and useful (for both their CYP and schools’ objectives) to implement it [52, 58].
- Securing school participation requires good working relationships, knowledge, persistence, and passion from those ‘selling’ it.
- The importance of negating logistical issues (or instilling confidence in the ability to negate issues) is an important facilitator to the implementation of school screening tools [12, 15, 59, 60].
- Having a dedicated and influential school lead for establishing support, maintaining implementation, and managing logistics is crucial [51, 52].
- A variety of different strategies (e.g., using school leaders to encourage other schools to participate, presenting case studies of successful outcomes and how implementation barriers have been overcome) can help in securing school participation. The formalisation or consistent presentation of these strategies could be a useful way to encourage uptake of screening programmes.

### Study strengths and limitations

This is the first qualitative evaluation of a novel school-based, digital screening tool (the DHC), and the first to explore its potential for detecting and supporting unmet mental and physical health need in CYP. Our findings reflect the perspectives and experiences of a variety of key stakeholders. It is important, however, to acknowledge potential limitations of our study. First, we experienced difficulties in recruitment during the Covid-19 pandemic. While we attempted to recruit as wide a sample as possible, but recruitment was marred through data collection occurring during Covid-19 restrictions with, for example, social distancing requirements resulting in a lack of face-to-face team meetings to advertise participation. Participation information was disseminated throughout the PHSN teams and participating schools, with an open invite for participation. It is possible only those with more favourable perspectives volunteered to participate, with this biasing the findings. Whilst we were able to interview three school leaders involved in employing the DHC in the academic year, Covid-19 restrictions impacted upon uptake of the DHC more generally, meaning few schools were signed up to take part at the time of data collection, and issues around managing Covid-19 -related challenges meant many felt unable to offer time to participate. We were only able to capture the direct perspectives of schools that wanted to participate, and thus missed the perspective of schools who have not participated, despite contacting schools who had chosen not to (or felt unable to) participate’. In an attempt to redress this, we ensured that we explored PHSN perspectives about why some schools chose not to participate and their understandings of barriers to participation from the school perspective. Nevertheless, this provided extremely useful insights into the challenges and successes of those ‘selling’ the DHC to schools. Overall, our findings highlight important considerations that could be used to help enable the development, delivery, and wider implementation of the DHC and similar programmes. Therefore, despite challenges in recruitment, we were able to capture a broad and insightful range of perspectives from professional practitioners across different roles and contexts.

## Conclusions

The DHC, as a universal school-based screening programme which has linked follow-up intervention, has great potential to identify unmet health need and result in better health outcomes for CYP. Whilst the research context of this study may not be representative of all wider contexts, the perspectives and experiences of those involved in delivering the DHC highlight important considerations which may enable effective implementation and delivery of school screening programmes across other areas.

## Supplementary Information

**Additional file 1.**

**Additional file 2.**

**Additional file 3.**

**Additional file 4.**

## Data Availability

The qualitative datasets generated and/or analysed during this study are available from the corresponding author on reasonable request, and subject to approval from the School of Health and Related Research (ScHARR) ethics committee at the University of Sheffield. .

## Abbreviations

CAMHS: Child and Adolescent Mental Health Services
CYP: Children and young people
DHC: Digital Health Contact
NHS: National Health Service
NICE: National Institute for Health and Care Excellence
Ofsted: Office for Standards in Education, Children’s Services and Skills
PHSN: Public Health School Nurses
PSHE: Personal, social, health and economic education
SLT: Senior Leadership Team

## Declarations

CM and MC are employed at the DHCs commissioning organisation (at Leicester City Council), and ST and LH are employed at the DHCs provider organisation (at Leicestershire Partnership NHS Trust).

This study, and the wider research project evaluating the DHC, has been co-produced with the DHC’s commissioners (at Leicester City Council) and providers (at Leicestershire Partnership NHS Trust). The commissioner and the provider are committed to helping to facilitate a robust evaluation of the DHC in order to inform potential changes and wider roll out of the programme. There has been no pressure or influence to modify, restate, weaken, omit or frame findings, conclusions and recommendations from any team member.
