# Supplementary material for: Exploring the potential of a school-based online health and wellbeing screening tool: professional stakeholders’ perspectives and experiences
Source: BMC Public Health. 2022 Feb 16;22:324. doi: 10.1186/s12889-022-12748-2 (PMC8848969; doi:10.1186/s12889-022-12748-2)
Supplement: Supplementary file 1 — Additional file 1. [file 12889_2022_12748_MOESM1_ESM.docx]

Topic guide: **Provider and Commissioners**

Note: This topic guide is indicative and will be subject to incremental change from emergent findings/information during the research. This is a guide to the topics to be covered during the interviews, it is not a script, and therefore the order of topics will be flexible.

Introduction:

- Thank you for participating
- Introduction of self
- introduction of study
- Key points
  - length of interview (60 minutes)
  - interview as a discussion which will cover key topics
  - no right or wrong answers - exploring perspectives
  - participation is voluntary - rights around withdrawing participation
  - confidentiality/anonymity
  - how findings will be reported
  - interview will be audio recorded
  - Questions? Happy to proceed? Complete and sign consent form

RECORD [obtain verbal consent as telephone interview]

Participant role/background

- Clarify current role and responsibilities
- Organisation (purpose and activities)
- role in relation to DHC
- Experience of the DHC programme

Digital Health Contact (DHC)

Purpose of DHC

- Why was it established / what was the rationale
- What are the priory outcomes from the programme?
  - what do you hope the programme achieves

Can you provide an explanation of how the DHC runs?

- how is it presented to schools
  - what is the process of setting up school participation
- When does data collection start
  - is it different for different school
- when does analysis occur
- when do interventions start
- when do school get feedback
  - what does this feedback look like
  - what information is shared with schools - how is this decided
- How well do you think the programme is working
  - differences between how it runs in theory and practice
  - Is there consistency in how the programme is implemented (between schools)
  - What are the time/resources required for the DHC?
    - Is the programme an effective use of time/resources
    - are there competing needs / uses of resources
      - What (if anything) could have been provided instead of the DHC?
- What have been some of the implementation issues of the programme?
  - Wider challenged of health education and promotion in schools
  - Are there any conflicts within schools between the DHC as a ‘health intervention’  and the educational priorities/agendas of school

How has the programme changed and developed?

- are the original aims of the programme still the same
- what learning has the current pilot provided - and how has this been used

How have the questions/topics been devised?

- How were the ‘red flag’ questions developed
- How were the ‘red flag’ ‘trigger’ words established
  - consultation with specialist services? Young people?
  - based upon regional need/issues

Was there an anticipated level of ‘red flag’ results being returned from the DHC?

- how does this reflect the initial findings from the pilot
- Has the number of ‘red flag’ results at each stage of the programme (following survey, initial triage, those offered interviews) prompted any change in the DHC

How does the referral process following the program work?

- are there clear and consistent referral pathways to services around this
  - are there any issues with these pathways (waiting lists, thresholds)
    - If so, how are these dealt with?
- Is there capacity and ability to meet any ‘unmet’ health need?
  - within school
  - by specialist services

Reflections and future plans

Is the data being returned useful?

- What has/is the data being used for?

What could be improved in relation to the project?

- what are the current issues
  - how could these be overcome
  - difficulties in changing current practices
- If starting the project again, what would be done differently

What are your future plans/intentions around the project?

- do you think the DHC programme could be transferred to different areas
  - what would be barriers to this

**Thank and finish**

- anything else they would like to add
- any suggestions for further participants
- any key documentation that the study team would find useful

Topic guide: **School leaders**

Note: This topic guide is indicative and will be subject to incremental change from emergent findings/information during the research. This is a guide to the topics to be covered during the interviews, it is not a script, and therefore the order of topics will be flexible.

Introduction:

- Thank you for participating
- Introduction of self
- Introduction of study
- Key points
  - length of interview (60 minutes)
  - interview as a discussion which will cover key topics
  - no right or wrong answers - exploring perspectives
  - participation is voluntary - right to withdraw participation
  - confidentiality/anonymity
  - how findings will be reported
  - interview will be recorded
  - Questions? Happy to proceed? Complete and sign consent form

RECORD [obtain verbal consent as telephone interview]

Participant role/background

- current role and responsibilities
- Role in relation to DHC
- Experience in the DHC programme

Digital Health Contact (DHC)

Brief explanation of DHC (your understanding)

- how does it work/run
  - timeline - when is data collected, when do schools receive feedback
- How has the DHC been explained to you, by whom
- what does the programme do
  - what do you know about the health education and promotion aspect of the DHC

Motivations for participation

- What was the schools motivation for involvement of the programme
  - current motivations
  - What were your motivations for involvement
- what benefits do you perceive from engagement in the programme
  - for: school - young people - public health
  - in the short and long term?
  - do you perceive both short and/or long term benefits
  - any negatives
- how useful do you see the programme
  - for identifying need
  - for health education/promotion

What data does the school receive from the DHC?

- outcome of questionnaire - number of red flags
- follow up appointments (initial and support sessions)
- How are the findings of the DHC used in the school
  - implemented support / work packaged within school/classes?
- How useful are the findings for the school?

How effective do you think the programme is in detecting  unknown and undisclosed health and wellbeing support needs for young people?

- - Do you think the DHC is picking up young people that the school was not previously aware of having health needs?
  - how could those with such ‘unmet’ needs be better picked up
  - Do you think those without ‘unmet’ needs are being picked up
    - what could be done to avoid this
  - Can you think of any alternative approaches/methods to support young people to disclose any health issues they might be experiencing?

How effective do you think the programme is in providing support for the needs it identifies?

- What is your understanding of what support / health advice/information the programme provides?
  - information and signposting after online survey
  - support provided from nurses
  - referral for other support
- What is your understanding about how ‘red flagged’ young people are assessed and supported
- How does the referral process following the program work?
  - what is the school's involvement in referrals / knowledge of young people being referred
- are there clear and consistent referral pathways to services around this
- Is there capacity and ability to meet any ‘unmet’ health need?
  - within school / by specialist services
- Has the identification of need in young people resulted in any unanticipated issues for the school?
  - how has this been dealt with

Reflections and future plans

What are the costs of providing the DHC?

- Does the DHC provide value for money
- what does ‘value’ mean for you
- was cost a consideration when deciding to participate

What is working well in relation to the programme?

- useful/positive data being returned

What could be improved in relation to the programme?

- what are the current issues
  - how could these be overcome
  - difficulties in changing current practices
- How well is the programme followed in school
  - are all children targeted/sampled
  - are the groups of young children ‘missed’ - is there a ‘mop-up’ survey?
- Does the school participate in other health initiatives?
          - are these complimentary with the DHC programme
          -      how does the DHC compare with other approaches
  - Where do you think the DHC fits into the public health/local authority offer on health and wellbeing
- Is there opportunity and clear communication lines to discuss the programme with the providers

What are your future plans/intentions around the project?

- are you intending to continue to use the programme

**Thank and finish**

- anything else they would like to add
- any suggestions for further participants
- any key documentation that the study team would find useful

Topic guide: **Public Health Nurses, Healthy Child Programme Nurses, Healthy Child Programme Practitioners, Healthy Child Programme Support Workers**

Note: This topic guide is indicative and will be subject to incremental change from emergent findings/information during the research. This is a guide to the topics to be covered during the interviews, it is not a script, and therefore the order of topics will be flexible.

Introduction:

- Thank you for participating
- Introduction of self
- Introduction of study
- Key points
  - length of interview (60 minutes)
  - interview as a discussion which will cover key topics
  - no right or wrong answers - exploring perspectives
  - participation is voluntary - right to withdraw participation
  - confidentiality/anonymity
  - how findings will be reported
  - interview will be recorded
  - Questions? Happy to proceed? Complete and sign consent form

RECORD [obtain verbal consent as telephone interview]

Participant role/background

- current role and responsibilities
- Organisation’s purpose and activities

Digital Health Contract (DHC)

What is your understanding of the programme?

- what is its purpose/aims
- What is your understanding of your role in relation to DHC

Can you provide an explanation of how the DHC runs? (timeline)

- What is your involvement in presenting to/negotiating with schools
  - meeting teachers / pupils
  - common questions from schools
- Can you tell me about getting schools to participate
  - - issues / challenges
    - what do schools want/expect from involvement
- When does data collection start
  - what is your involvement in collecting data
  - How much time is spent collecting data?
- How long does assessing the red flagged young people take
  - how do you go about making decisions at the triage stage of those ‘red flagged’
- When do initial interviews take place
  - what are decisions to offer initial interview based on
  - How long between online survey and initial interview
  - What are decisions not to offer an assessment based on?
    - your capacity
    - thresholds of need
    - unnecessary red flags
  - if refuses interview what is done
- when do support session start / last
- how do you go about making decisions for onward referral

How effective do you think the programme is in detecting unknown and undisclosed health and wellbeing support needs for young people?

- do you think any aspects (online survey, interviews with nurses) are effective in young people disclosing unmet health needs
  - what could be more effective / alternative approaches
- Do you think there are aspects of the DHC process (how the programme runs) which may result in ‘unmet’ needs being missed
  - young people who parents opt them out
  - young people refusing interview
  - young people not taking part in survey (expelled/excluded, partial timetable, absent)
  - Are there other health-related issues missed by the DHC?
- Can you think of any alternative approaches/methods to support young people to disclose any health issues they might be experiencing?

What do you think about the sensitivity of the DHC (the number of ‘red flags’ picked up)

- - number of young people with a preliminary ‘red flag’ - high/low
  - could the DHC be changed to pick-up young people with unmet need and avoid picking-up those with without unmet need
    - how could this be done

How effective do you think the programme is in providing support for ‘unmet’ needs

- Can you talk about the health promotional elements of the DHC
  - health promotion at different stages of the programme
  - What health promotion/information resources are used
  - How was the inclusion of these different resources decided upon
    - who was involved in this - role of school nurses/HCP team
- packages of work delivered by nurses
  - any training/support around delivering follow-up session topics (i.e., from specialist services) due to wellbeing issues being diverse
    - details of training if relevant
  - is there capacity to deliver support
  - How well prepared do you feel delivering project generally
    - training
    - information on DHC / reasons for DHC
- referral to ‘specialist services’
  - Are there any issues translating needs into services (e.g. waiting lists, thresholds)
  - what is your involvement following referral

Delivering to young people / parents/guardians

How do you explain the DHC to children / parents

- frequently asked questions from children / parents

What has been your experience of disclosing redflags to children / parents

- how do they respond

How do you avoid disclosing red flags to the young people’s peers

- how are appointments for young people made and sessions delivered to avoid disclosing to others

Reflections

From your perspective is the DHC useful?

- in detecting unmet health needs and providing support

Is the programme an effective use of your time

- what other priorities do you have
- how do you balance these
- What would you be doing if you were not facilitating the DHC?
- What would you say are the costs to you?
  - time cost - administration/providing sessions

What is working well in relation to the programme?

- do you think there are particular groups of young people the DHC works best for

What could be improved in relation to the programme?

- any current issues
  - how could these be overcome

**Thank and finish**

- anything else they would like to add
- any suggestions for further participants
- any key documentation that the study team would find useful
