# Supplementary material for: Exploring the potential of a school-based online health and wellbeing screening tool: professional stakeholders’ perspectives and experiences
Source: BMC Public Health. 2022 Feb 16;22:324. doi: 10.1186/s12889-022-12748-2 (PMC8848969; doi:10.1186/s12889-022-12748-2)
Supplement: Supplementary file 2 — Additional file 2. [file 12889_2022_12748_MOESM2_ESM.docx]

**DHC Screening Questions**

Below is each question in the DHC screening questionnaire, and the responses to each question.

A follow-up questions and text box for open written responses accompanies each question if an answer suggests potential risk/concern.

Each question contains further information to aid comprehension.

1. Do you normally feel safe at home?

[*Very Safe – Safe – Okay – Unsafe – Very Unsafe*]

- 1. [*if answered ‘Unsafe – Very Unsafe’*] Why do you feel this way?

1. Do you normally feel safe at school?

[*Very Safe – Safe – Okay – Unsafe – Very Unsafe*]

- 1. [*if answered ‘Unsafe – Very Unsafe’*] Why do you feel this way?

1. Do you feel safe at your new school? (Year 7 only)

[*Very Safe – Safe – Okay – Unsafe – Very Unsafe*]

- 1. [*if answered ‘Unsafe – Very Unsafe’*] Why do you feel this way?

1. Are you being bullied at school?

[*Yes – No*]

- 1. [*if answered ‘Yes’*] What type of bullying are you experiencing physical/verbal/cyber)?
  2. Have you told an adult at school about this? [*Yes – No*]
  3. Are you receiving support for this? [*Yes – No*]

1. Have you got any worries about your physical health?

[*Yes – No*]

- 1. [*if answered ‘Yes’*] Please provide more information about your concerns?

1. Do you have any health needs or disabilities that prevent you from attending school or learning during your lessons?

[*Yes – No*]

- 1. [*if answered ‘Yes’*] Please provide more information about your concerns?

1. Do you feel safe online?

[*Yes – No*]

- 1. [*if answered ‘Yes’*] Please provide more information about your concerns?

1. Have you got any worries about exams? (Year 9 & 11 only)

[*Yes – No*]

- 1. [*if answered ‘Yes’*] Please provide more information about your concerns?

1. Do you regularly feel frightened, worried or nervous?

[*Yes – No*]

- 1. [*if answered ‘Yes’*] Do you have someone to talk to about this?

1. Do you self-harm?

[*Yes – No*]

- 1. [*if answered ‘Yes’*] Are you currently receiving support from an adult?

1. Do you regularly feel low in mood? You might think about this as feeling sad.

[*Yes – No*]

- 1. [*if answered ‘Yes’*] Are you currently receiving support from an adult?

1. Have you got any worries about your appearance/body image?

[*Yes – No*]

- 1. [*if answered ‘Yes’*] Please tell us more about your concerns?

1. On average, how many hours sleep do you get each night?

[*under 6 hours – between 6 and 7 hours - between 7 and 8 hours - between 8 and 9 hours - between 9 and 10 hours – Over 10 hours]*

1. Have you got any worries relating to body changes/puberty?

[*Yes – No*]

- 1. [*if answered ‘Yes’*] Please provide more information about your concerns?

1. Do you have any worries about your sexuality? (Year 9 & 11 only)

[*Yes – No*]

- 1. [*if answered ‘Yes’*] Please provide more information about your concerns?

1. Do you have any worries about your diet?

[*Yes – No*]

- 1. [*if answered ‘Yes’*] Is this linked to (not eating breakfast – not having enough fruit and vegetables – frequent takeaways – not having enough to eat – something else)
  2. [*for any response*] Please provide more information about your concerns?

1. Do you exercise for 60 minutes a day, 5 days a week?

[*yes – no*]

1. Do you help to look after someone in your household every day who fits one of the below descriptions?

[*sick – disabled - has mental health problems - uses alcohol and/or drugs – I don’t look after anyone*]

1. Do you drink alcohol?

[*Yes – No*]

1. Do you use drugs that have not come from a doctor or chemist?

[*Yes – No*]

1. Currently, do you smoke?

[*Yes – No*]

- 1. What do you smoke? (cigarettes – shisha – e-cigarettes)

1. Have you been in or are you currently in a sexual relationship? (Year 9 & 11 only)

[*Yes – No*]

1. Are you worried that Child Exploitation (CE) is happening to you or someone you know?  (Year 9 & 11 only)

[*Yes – No*]

- 1. [*if answered ‘Yes’*] What are you worried about? (sexual exploitation – drugs – carrying knives/weapons – forced labour – something else)
  2. Please provide more information about your concerns?

1. Did you know that you should check your breasts/testicles every month? (Year 11 only)

[*Yes – No*]

1. Have you had an eye test in the last 12 months?

[*Yes – No*]

1. Have you been to the dentist in the last 12 months?

[*Yes – No*]

1. Do you know that forced marriage is illegal in the UK?

[*Yes – No*]

1. Do you know that Female Genital Mutilation (FGM) is illegal in the UK?

[*Yes – No*]

1. Do you understand what extremism is?

[*Yes – No*]

1. Do you know where you can get help or advice for your health when you leave school? (Year 11 only)

[*Yes – No*]
