# Supplementary material for: Exploring the potential of a school-based online health and wellbeing screening tool: professional stakeholders’ perspectives and experiences
Source: BMC Public Health. 2022 Feb 16;22:324. doi: 10.1186/s12889-022-12748-2 (PMC8848969; doi:10.1186/s12889-022-12748-2)
Supplement: Supplementary file 3 — Additional file 3. [file 12889_2022_12748_MOESM3_ESM.docx]

DHC Stakeholder coding framework

| **Theme** | **Sub-theme** |
| --- | --- |
| Understandings of the DHCs purpose | Perceived Aims/objectives/rational  Received explanation of DHC |
| Understandings of the DHCs process | Delivery timeline  Nurse Triaging process  Assessment and support sessions  Onward referral to specialist services |
| Perceived benefits | Motivation for school involvement/participation  Improving knowledge of / access to PHSN and support services  Perceptions of effectiveness/efficiency  Ability to detect ‘unmet’ need  Individual/school level data shaping work/targeting resources |
| Implementation barriers/facilitators | Presentation/’selling’ to schools  Relationships between schools and PHSN  Resource requirements (time, staff)  Logistics of set-up and delivery |
| Development of the DHC | Question development/refining  Health promotion information/material |
| Suggested improvements | School implementation  Better detecting need |
| Impact of Covid-19 upon programme delivery |  |
