# Supplementary material for: Exploring the potential of a school-based online health and wellbeing screening tool: professional stakeholders’ perspectives and experiences
Source: BMC Public Health. 2022 Feb 16;22:324. doi: 10.1186/s12889-022-12748-2 (PMC8848969; doi:10.1186/s12889-022-12748-2)

Example of DHC questions from online screening questionnaire – showing how responses to questions result in a text box being provided for written information.


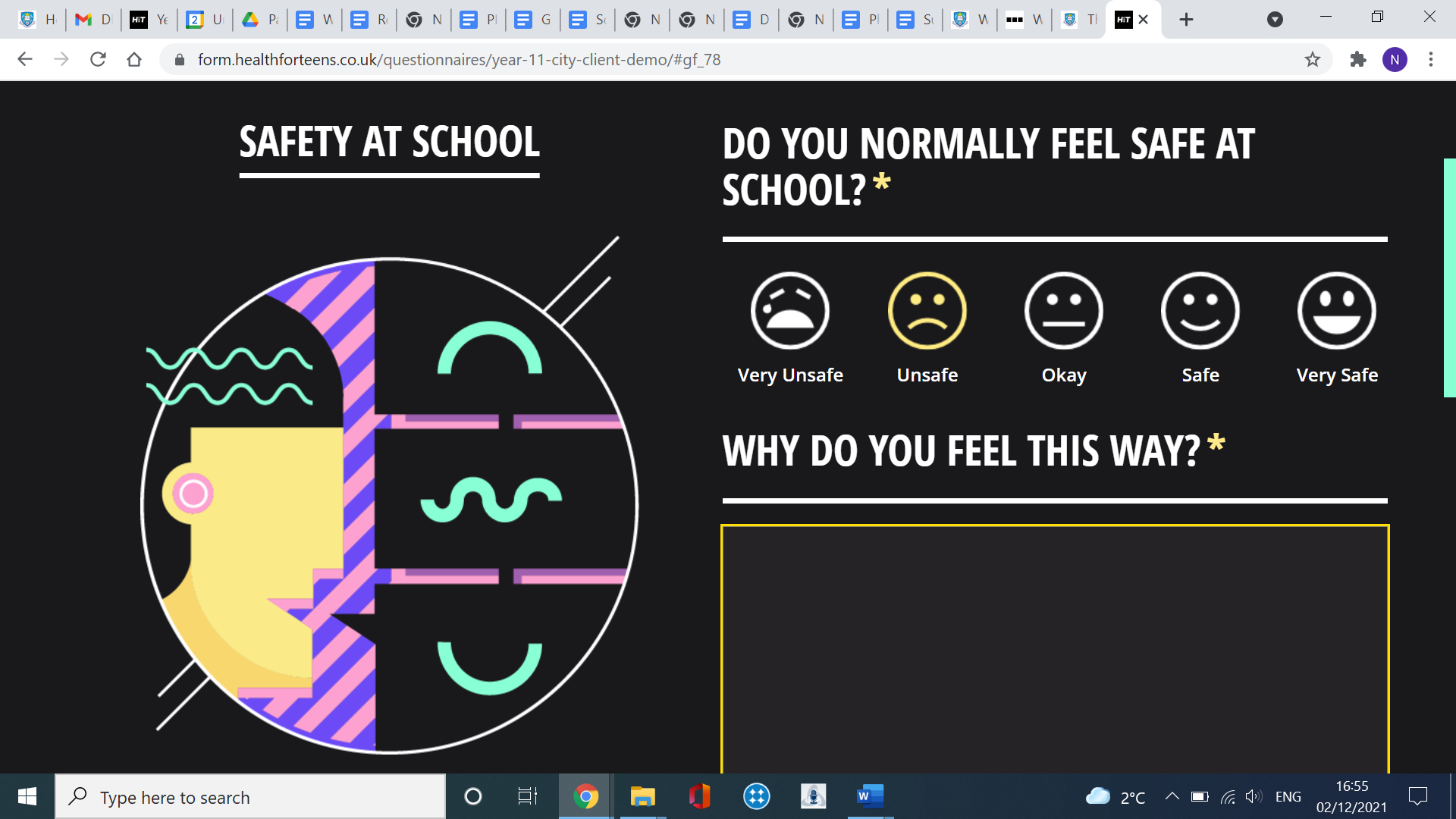


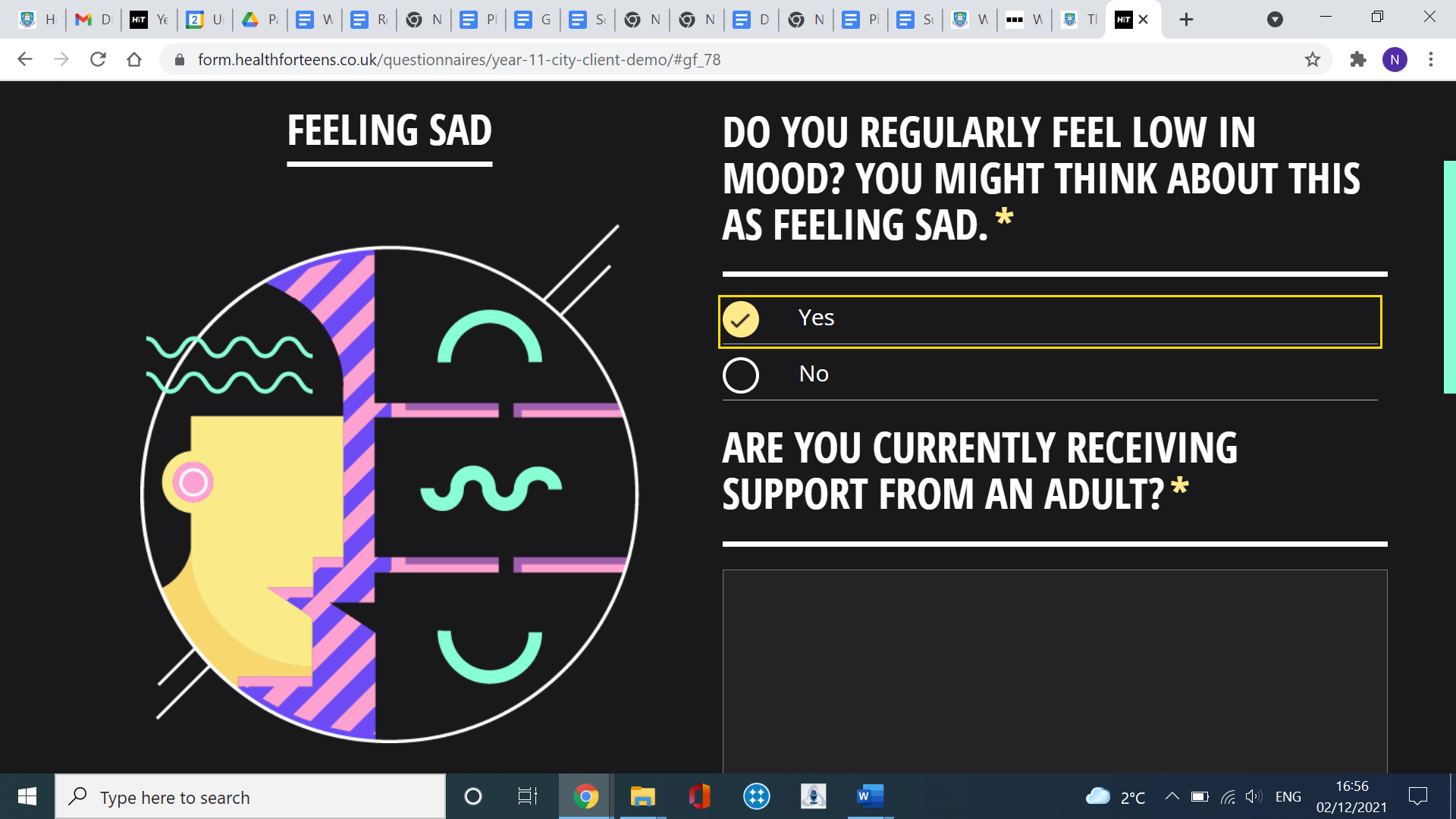

Supplement: Supplementary file 4 — Additional file 4. [file 12889_2022_12748_MOESM4_ESM.docx]
